# Vitamin D in pregnancy (GRAVITD) – a randomised controlled trial identifying associations and mechanisms linking maternal Vitamin D deficiency to placental dysfunction and adverse pregnancy outcomes – study protocol

**DOI:** 10.1186/s12884-023-05484-x

**Published:** 2023-03-15

**Authors:** Anna Louise Vestergaard, Martin Christensen, Mette Findal Andreasen, Agnete Larsen, Pinar Bor

**Affiliations:** 1 Department of Obstetrics and Gynecology, Randers Regional Hospital, Østervang 54, 8930 Randers NØ, Denmark; 2 Department of Clinical Medicine, Aarhus University, Aarhus, Denmark; 3 Department of Forensic Medicine, Section for Forensic Chemistry, Aarhus University, Aarhus, Denmark; 4 Department of Biomedicine, Aarhus University, Aarhus, Denmark

**Keywords:** Vitamin D, Pregnancy, Pre-eclampsia, Foetal growth restriction, Gestational diabetes, Placenta

## Abstract

**Background:**

The prevalence of vitamin D deficiency is high among pregnant women. Vitamin D deficiency in pregnancy is associated with increased risk of adverse pregnancy outcomes especially complications related to placental dysfunction and insulin resistance. The objective of this study is to investigate if a higher dose of vitamin D supplementation in pregnancy reduces the prevalence of vitamin D deficiency and prevents adverse pregnancy outcome with special emphasize on preeclampsia, foetal growth restriction and gestational diabetes.

**Methods:**

GRAVITD is a double-blinded randomised trial with parallel groups where all pregnant women attending the free of charge national nuchal translucency scan programme in gestational week 10–14 at Randers Regional Hospital are invited to participate. Enrolment started in June 2020. Participants are randomised in a two armed randomization with a 1:1 allocation ratio into 1) control group – receives 10 µg of vitamin D or 2) intervention group – receives 90 µg of vitamin D. A total of 2000 pregnant women will be included. Maternal blood samples and questionnaires describing life-style habits are collected upon enrolment. For half of the participants blood samples and questionnaires will be repeated again in 3rd trimester. Blood samples will be analysed for 25-hydroxy-vitamin D using high-performance liquid chromatography coupled with tandem mass spectrometry. Upon delivery, placental tissue and umbilicalcord blood will be collected and information on maternal and fetal outcomes will be exstracted from medical records.

The primary outcomes are serum levels of 25-hydroxy-vitamin D ≥ 75 nmol/L and the rate of preeclampsia, foetal growth restriction and gestational diabetes. Secondary outcome includes identification and impact on placental functions related to vitamin D. A tertiary outcome is to initiate a cohort of children born from mothers in the trial for future follow-up of the effects of vitamin D on childhood health.

**Discussion:**

Provided that this trial finds beneficial effects of a higher dose of vitamin D supplementation in pregnancies, official recommendations can be adjusted accordingly. This will provide a low-cost and easily implementable adjustment of prenatal care which can improve health for both mother and child during pregnancy and beyond.

**Trial registration:**

ClinicalTrial.gov: NCT04291313. Registered February 17, 2020

## Background

Vitamin D deficiency is common among pregnant women worldwide with a reported prevalence of up to 92% in the most severely affected populations [1, 2]. Common causes include living in Northern countries at higher latitude and a darker skin tone [3–5]. Further, the growing prevalence of pregnant women with adiposity is disturbing in this context, as adiposity increases the risk of vitamin D deficiency [6]. Vitamin D status is a matter of concern in the clinical setting, as vitamin D deficiency appear to affect foetal growth trajectories, just as vitamin D deficiency is associated with an increased risk of pregnancy complications related to placental dysfunction. Vitamin D deficiency hence increases the risk of pre-eclampsia (PE), gestational diabetes (GDM), low birthweight and preterm birth [7–10]. Moreover, insufficient maternal vitamin D levels (25(OH)D) is associated with long-term health risks for the offspring [11]. In utero vitamin D exposure affects bone development and the strength of the tooth enamel [12, 13]. Previous studies also link exposure to low levels of vitamin D during intrauterine life to an increased risk of later disease including increased risk of asthma, type 1 diabetes, autism, schizophrenia, and multiple sclerosis in the offspring [10, 11, 14].

Since only 60–80% of maternal 25(OH)D is transferred to the foetus [15], the maternal serum 25(OH)D level needs to be ≥ 75 nmol/L to ensure that the foetus reaches a 25(OH)D level of at least 50 nmol/L. In earlier studies from Denmark, around 30% of pregnant women had aa 25(OH)D level < 50 nmol/L and as many as 70–88% of the pregnant women did not reach a serum 25(OH)D level > 75–80 nmol/L [5, 16] putting the foetus at risk of developing vitamin D deficiency. In a previous cohort study we characterized our present study population, i.e. women giving birth at Randers Regional Hospital, a hospital covering both urban areas, smaller cities and rural areas. Here we found that 10% of the participants had vitamin D deficiency (25(OH)D level < 50 nmol/L) and only 58% reached a sufficient 25(OH)D level of ≥ 75 nmol/L [17]. Notably, this was a study undertaken in the summer months and with a high adherence (86%) to the official Danish recommendations of a daily vitamin D supplement of 10 µg during the entire pregnancy. However, the officially recommended intake of 10 µg vitamin D daily is mainly based on a more than 35 year old small-scale study from Norway [18, 19] and does not take into account the nutritional differences between Denmark and Norway – e.g. a higher prevalence of fish consumption in Norway [20].

PE, one of the pregnancy complications most strongly associated with vitamin D status [7], affects 3–5% of all pregnancies worldwide and PE is still a major cause for maternal and perinatal morbidity and mortality [21, 22]. Danish registers find that 3% of all pregnancies are complicated by PE [23]. We found a similar but slightly higher prevalence in our previous study conducted in 2016–2017 as 4.1% developed PE [17].

In our previous study, around 10% of the children were born small for gestational age (SGA) (birthweight below the 10th percentile) [17]. If growth restriction occur during pregnancy this increases the risk of complications in the prenatal period [24], and also the risk of diseases later in life [24, 25]. Such increased risks for the child occur when the estimated weight of the foetus is -15% (i.e. < 10th percentile) below the appropriate weight for the gestational age (i.e. foetal weight between the 10th 90th percentile), the condition coined foetal growth restriction (FGR). The risk is even bigger if the weight of the foetus is -22% or more below appropriate weight, the condition coined intrauterine growth restriction (IUGR) [24]. In our previous study, we found that average vitamin D levels were reduced in women who had children with PE and IUGR albeit the difference were only significant in case of IUGR [17]. Such associations have also been found by others [9].

Today GDM is described as the most common metabolic disorder of pregnancy with an increasing prevalence, affecting around 7–10% of all pregnancies worldwide [26]. Moreover, the risk of developing GDM is correlated with increasing maternal age and a high pre-pregnancy BMI. These are factors that are also increasing globally among the pregnant population [27–30]. GDM is considered as an early marker of glucose intolerance, associated with both insulin resistance and impaired insulin secretion, thereby an increased risk of maternal and fetal adverse outcomes such as macrosomia, birth trauma, respiratory distress syndrome, jaundice, and hypoglycemia, an increased rate of delivery with cesarean section, preterm labor, FGR and PE. Furthermore, there is a higher risk of obesity and diabetes in later life both for these women and their children. Some studies suggested that vitamin D play a crucial role in maintaining normal glucose levels and reduces the extent of pathologies associated with insulin resistance [31, 32].

Although it is well known that vitamin D is important in pregnancy, there are still conflicting results on whether vitamin D supplementation can reduce the prevalence of pregnancy complications. In 2012, Hollis & Wagner [33] reported that an increased dose of vitamin D supplementation in pregnancy could improve birth outcomes and lead to a significantly decreased prevalence of hypertensive disorders in pregnancy in a randomised controlled trial. Similar results have since been found by others in relation to PE [34–37], GDM [37, 38] and foetal growth [34, 36], however others found no effect [39, 40]. One of the main weaknesses in previous trials is a small sample size leading to lack of statistical power. Compiling existing data is also troublesome due to a large variance in the method chosen for evaluation of 25(OH)D status in different study populations. Notably, significant differences occur among different methods for measuring 25(OH)D [41] and too few studies use the relatively expensive methods using high-performance liquid chromatography coupled with tandem mass spectrometry (HPLC–MS/MS) despite the fact that these methods are considered to be the gold standard for measuring 25(OH)D [41, 42]. Another challenge in previous studies with vitamin D is the administration and doses of vitamin D and the duration of the intervention, which differs a lot from study to study. Along with this, it is important to keep in mind when comparing different clinical studies that the study design should be comparable in order for the results to be comparable.

As vitamin D is generated in the skin, another major challenge when performing clinical studies is sunshine exposure, which varies a lot according to geography but also according to season [43]. This is also enhanced by differences in skin tone, which has a large impact on the ability to produce endogenous vitamin D in the skin. A well designed large randomised clinical trial, covering a long enrolment period to allow for seasonal variance, is lacking.

## Methods

### Study design/aim

The aim of this clinical trial is to investigate if a higher daily dose of vitamin D, than what is currently recommended in Denmark, can reduce the prevalence of the major pregnancy complications related to low vitamin D level in pregnancy, when initiated after pregnancy is determined (week 9 to 14).

The trial is a double-blinded randomised clinical trial with parallel groups and a two armed randomization with a 1:1 allocation ratio. Focus is on the pregnancy complications related to placental dysfunction – PE, FGR (≤ -15% of the appropriate weight for the gestational age) and GDM, in addition effects of placental function will also be investigated. Enrolment to the trial will take place over a 2.5–3-year time period covering both the summer and winter months in order to cover periods of different sunshine exposure and variance from year to year.

### Study settings

The trial is conducted at the Department of Gynaecology and Obstetrics, Randers Regional Hospital, Denmark.

### Participants

Pregnant women attending nuchal translucency scan in gestational week 11–14 as part of the national prenatal screening program at Randers Regional Hospital are invited to participate in the trial. The nuchal translucency scan is a part of the national prenatal screening program, which is offered to all Danish pregnant women and attended by 94% [44]. The screening program is free of charge. When they attend the nuchal translucency scan at the hospital they receive both orally and written information about the trial by a member of the research group. At Randers Regional Hospital there are around 2,200–2,400 childbirths annually. Women who agree to participate will give informed written consent before participation in agreement with The Declaration of Helsinki.

#### Exclusion criteria

- Age < 18 years
- Unable to give written informed consent
-  > 15 complete weeks pregnant at the time of the nuchal translucency scan
- Disturbances in calcium metabolism
- Chronic kidney disease
- Vitamin D treatment initiated by a physician

### Intervention

**Fig. 1:**
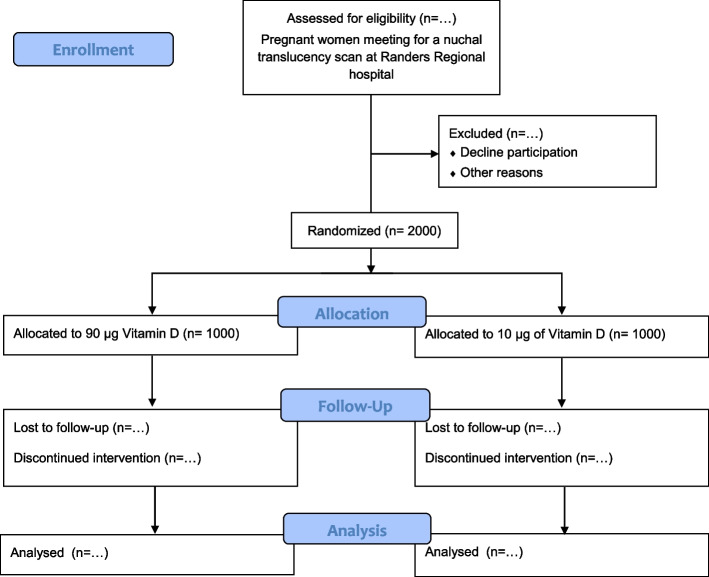
CONSORT flow diagram of the trial with intended numbers

All pregnant women who agree to participate will be randomised in equal proportions to receive either an extra vitamin D supplement (80 µg) or a placebo tablet. The participants will all receive a prenatal multivitamin (Livol®) containing 10 µg of vitamin D, which is the current national recommendation for pregnant women in Denmark [18, 45]. Thereby, all participants as a minimum gets the recommended vitamin D supplementation and are not in a higher risk of vitamin D deficiency than what they are at baseline and if they do not participate in the trial. There will be two parallel groups in the trial receiving either 90 µg or 10 µg of vitamin D respectively (Fig. 1). The dose chosen for the intervention group (90 µg) is within a dose interval previously proven safe by others [42, 46, 47] and the dose is 10% below the current limit for what can be purchased in Denmark without a prescription. The vitamin D supplement tablet and the placebo tablet are identical in appearance and taste and contain exactly the same ingredients except from the cholecalciferol which is only in the vitamin D supplement tablet. The vitamin D supplement and the placebo tablets are packed in anonymized containers and provided with a unique number. Vitamin D supplements, placebo tablets and the prenatal multivitamin (Livol®) are provided by Orkla Care, Denmark. Vitamins for the remaining part of the pregnancy will be handed out at enrolment and the women will be instructed to take the vitamins daily until delivery.

#### Adherence

In order to monitor adherence in the trial, all participants will receive an email with a three-item adherence questionnaire 168 days after inclusion to the trial, which is around gestational week 36–37. They are asked whether they take the supplements every day or if they have forgotten to take them either a few times or for a longer period of time. Further, they are asked if they take the project supplement and the prenatal multivitamin or just one of them. If the questionnaire is not returned a reminder will be sent out after five days and again after 10 days.

#### Concomitant care

The participants are not allowed to take other vitamin D containing supplements than those handed out as part of the intervention. At the time of enrolment participants will be thoroughly informed about this. Further, it is recommended that all participants continue to take a 40–50 mg iron supplement from week 10 in pregnancy (not handed out by the study group), as the recommendations for pregnant Danish women prescribe. The prenatal multivitamin handed out in this trial contains 27 mg of iron, but since this multivitamin also contains 400 mg of calcium, the iron in the tablet is poorly absorbed. Therefore, a recommendation to the participants will be to take a separate daily iron supplement and to ingest this supplement at least 3 h apart from the supplements handed out in this trial.

### Outcome

#### Primary

- The incidence of PE
- The incidence of FGR (defined as a negative deviation of more than 15% (10th percentile) from the expected foetal weight diagnosed at ultrasonography scans)
- The incidence of GDM (defined as 2-h plasma glucose ≥ 9 mmol/l following a 75 g oral glucose load)
- Maternal serum-25(OH)D > 75 nmol/L

#### Secondary

- Birthweight
- Size related to gestational age (Small for Gestational Age; SGA, Appropriate for Gestational Age; AGA, Large for Gestational Age; LGA)
- The incidence of preterm birth (birth < 37 weeks of gestation)
- The incidence of postterm birth (birth > 40 weeks of gestation)
- The incidence of gestational hypertension (blood pressure > 140/90)
- Mode of delivery
- The incidence of infection during delivery
- Use of antibiotics during labour
- Admission rate to the neonatal ward
- The incidence of postpartum haemorrhage (bleeding > 500 ml)
- APGAR score at 1, 5 and 10 min after delivery
- Identification of placental functions related to maternal vitamin D status especially those also related to vitamin D metabolism and pregnancy-complications

#### Tertiary

Establishment of a new cohort of children born of mothers in the trial for future follow-up in order to investigate the effect a higher prenatal vitamin D supplementation has on the off-spring.

### Participant timeline

**Fig. 2:**
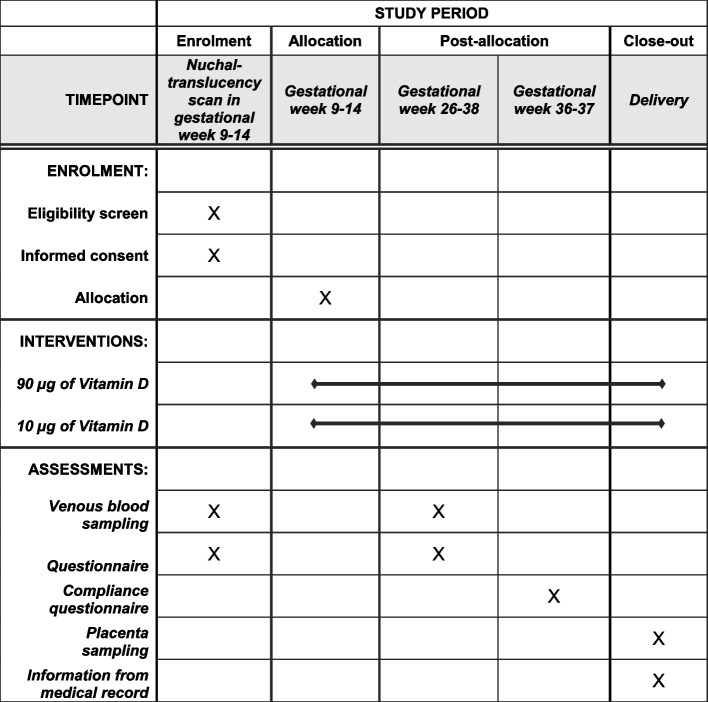
SPIRIT timeline table for the GRAVITD trial

Figure 2 shows the participant timeline.

### Randomisation and blinding

Randomisation is done in a 1:1 ratio, using Research Randomizer (version 4.0). To avoid skewering of data randomisation is done for 200 numbers a time. To assure an even distribution in the two intervention groups trough out the year to secure even distribution of season and hence sunshine exposure, the project supplements are packed in batches of 100 containers with 50 containers with placebo and 50 containers with vitamin D supplements. This is being repeated 10 times in total (n = 2,000). The containers with vitamin D supplement and those with placebo are identical and they are all marked with a unique number referring back to the randomisation. When a woman is included in the trial she will get a random container with tablets. The intervention is blinded for the participants and for all health professionals treating the women during their pregnancy and delivery. Only certain members of the research group, with no role in treatment and diagnosing the women, have access to the master list of randomisation numbers. Unblinding is permissible if a participant develops signs of Vitamin D intoxication and hypercalcemia.

### Data collection methods

#### Blood samples

Venous blood samples will be collected at inclusion. For half of the participants a venous blood sample will be collected in their 3rd trimester as well. Blood samples will be analysed for 25(OH)D at Department of Clinical Biochemistry, Aarhus University Hospital, Denmark using HPLC–MS/MS, which is considered as the gold standard method for measurement of 25(OH)D [41]. Blood samples will also be analysed for calcium, phosphate, iron, zinc and HbA1c at Department of Clinical Biochemistry, Randers Regional Hospital, Denmark.

#### Questionnaires

At inclusion all participants will fill out a questionnaire together with a member from the study group. The questionnaire contains questions about demographic, different aspects of lifestyle (diet, use of dietary supplements, smoking, caffeine intake and alcohol consumption), use of medication, acute- (including covid-19), chronic and genetic diseases, earlier pregnancies, fertility treatment and some pregnancy discomforts (morning sickness, constipation). The questionnaire has a section devoted to sunshine exposure where participants are asked about travel outside of Denmark, sun tanning, use of sunscreen and use of artificial sun.

The participants who return for a blood sample in their 3rd trimester will fill out a new questionnaire containing the same questions about lifestyle, use of medication, diseases and sunshine exposure and some more questions about their current pregnancy.

The questionnaires used in the trial can be obtained by contacting the corresponding author.

#### Biological samples from delivery

At delivery, the midwife will take blood samples from the umbilical cord and store the placenta at 5 °C. A member of the study group will afterwards perform systematic sampling of the placental tissue. After informed consent from participants, placenta samples are either placed in RNAlater or snap frozen and stored at -80 °C for future biological studies on genes and proteins related to vitamin D metabolism. Next Generation RNA Sequencing will be performed on a subgroup of placenta samples.

#### Information from medical records

The following data will be collected from medical records:

- Regarding pregnancy: duration of pregnancy, blood pressure (in 1st, 2nd and 3rd trimester, at delivery and after delivery), traces of glucose and protein in urine samples, the placement of the placenta, fetal growth and pregnancy related diseases.
- Regarding delivery: mode of delivery, need of initiation of labour, need of oxytocin stimulation, use of analgesia, pyrexia during labour (defined as ≥ 38.2 °C with epidural, without epidural: ≥ 38 °C), use of antibiotics during labour, estimated volume of blood loss at delivery and 2 h postpartum, placental weight and need for caesarean section or assisted delivery.
- Regarding the new-born: birthweight, sex, APGAR score at 1-, 5- and 10 min, admission to the neonatal ward, neonatal infection and malformations.

#### Data management

All data will be registered in an electronic case report form designed for the trial using the Research Electronic Data Capture (REDCap) database. The data collection form can be obtained by contacting the corresponding author.

### Statistics

#### Power calculation

There is no current information on what effect an increased vitamin D intake will have on the prevalence of preeclampsia or any of the other pregnancy complications of interest. Hence we have based our power calculation on a hypothesis of a 50–55% reduction in the prevalence of PE which we except to be the least common of the main targets, PE, FGR and GDM.

We expect a prevalence of PE of > 4% among the pregnant women getting the 10 µg vitamin D based on a prevalence of 4.1% in our previous study [17] with inclusion mainly during the summer months, hence the prevalence of PE will probably be a little higher when inclusion is done during both winter and summer months.

With a presumed prevalence of PE at > 4%, a significance level of 0.05 and a power of 80% then 1,000 women in each group will be sufficient to show a statistically significant difference between the two interventions despite a dropout of 5%. The entire study sample will be 2,000 women.

To test the hypothesis, that all participants in the intervention group reach a sufficient 25(OH)D level (≥ 75 nmol/L), a power calculation show that 15 women are needed in each group. The calculation is done with a power of 80% and a significance level of 0.05 and a presumed prevalence of 42% vitamin D insufficiency (25(OH)D < 75 nmol/L) before the intervention, based on data from our previous study [17]. Hence, we have chosen to take a second blood sample in the 3rd trimester in half of the cohort, resulting in 500 in each group.

#### Statistical analysis

Data will be analysed according to intention to treat, but if there is a non-compliance and dropout of more than 10%, we will also perform the statistical analysis without those with non-compliance. Demographic data will be presented as counts and percentages for categorical variables and continuous variables will be presented as mean and standard deviation. Data will be tested for normality using QQ-plots and will be log transformed if applicable. For all statistical analysis a level of statistical significance will be defined as 0.05. The primary outcomes PE, FGR and GDM, will be assessed by comparing the event rates in the two groups using a chi-square test and results will be presented as absolute and relative risks along with 95% confidence intervals (Cl). A similar approach will be used for categorical secondary outcomes. A multiple linear regression model will be used to adjust for indifferences in baseline characteristics. The 25(OH)D level will be assessed by comparing the mean in the two groups using students t-test. The same approach will be used for continuous secondary outcomes. Linear regression models will be used to assess continuous variables such as serum levels, blood pressure and biological changes in the placentas.

Subgroup analysis will be performed for the following subgroups:

- 25(OH)D level at enrolment (< 50, 50–75, > 75)
- Parity (nulliparous vs primiparous and multiparous)
- Ethnicity
- BMI (Body Mass Index) (≥ 20- < 25, ≥ 25- < 30, > 30, > 35, > 40)
- Extreme weight gain during pregnancy
- Smoking status
- Relevant medication
- Socio economic status (income and education)

### Harms/side effects

The vitamin D dose used in this trial is a safe dose [48], and even higher doses of 100 µg/day (4,000 IE/week) or even 875 µg/week (35,000 IE/week) have previously been tested in pregnant women and were found safe [42, 46, 47]. A vitamin D supplement, containing the same amount of vitamin D as our vitamin D tablet, is in Denmark widely accessible to purchase without a prescription. It lies 10% below the upper limit recommended for pregnant women, so if a participant takes another vitamin containing 10 µg of vitamin D despite being told not to, she would not be in risk of taking too much vitamin D.

No side effects are expected with this dose, but the participant will be instructed to discontinue the intervention if she develops symptoms that could be a result of vitamin D intoxication followed by of hypercalcaemia (nausea, vomiting, stomach pain, muscle fatigue, bone pain, tiredness, confusion, depression, loss of consciousness) and there is no other reason for the symptoms to occur or a 25(OH)D level above 220 nmol/L is measured in a blood sample at any time during the study period.

## Discussion

The current recommendation regarding vitamin D in pregnancy in Denmark is based on a few small and older observational studies from other Northern countries with a diet that differs from the typical Danish diet when it comes to vitamin D containing food items such as fatty fish and the use of fish oil supplements. Since this recommendation was made a lot of new evidence have indicated that vitamin D has several additional beneficial effects in pregnancy than just bone development. New recommendations are therefore needed to fit the pregnant population today with an emphasize on the dietary habits, ethnicity and health status of today's population.

With this randomised trial it will be possible to determine if a higher intake of vitamin D in pregnancy can improve the health for pregnant women and their children, by preventing maternal and neonatal complications also associated with health risks in later life. The trial is designed with a statistical power to determine the effect of a higher vitamin D supplement on the risk of developing the major obstetric diseases related to placental dysfunction – PE, FGR and GDM. Such diseases can possibly be prevented for some women by taking an extra vitamin D supplement in pregnancy – a period where most women are already vigilant when it comes to their diet and use of supplements. Further, the trial is large enough to establish if all pregnant women need to take more vitamin D to avoid deficiency or if only some groups of pregnant women need higher doses of vitamin D in their pregnancy e.g. women suffering from overweight or obesity.

Our trial has some limitations. We reach the pregnant women at their first contact to the hospital, hence they are in gestational week 9–14 when enrolled in the trial and starting the intervention. As this is still several weeks before complications like PE, GDM and FGR occur, we would argue that this is not too late in pregnancy to initiate the intervention with a higher dose of vitamin D supplementation. Initiation of the intervention already before conception would most likely lead to a better effect of extra vitamin D. However, such a trial design would demand a vastly larger study population as the drop-out and lost-to-follow-up would be much higher. Furthermore, it would be challenging to reach enough women before conception and have them agree to participation in a trial. Therefore, such a trial design would be much more expensive and difficult to conduct, and a trial design like ours is a good alternative as initiation of the intervention is still in the first third of the pregnancy. Another limitation to the trial is the lack of skin type measurement. Skin type could have been evaluated using the Fitzpatrick visual scale, however as this is a visual scale it has certain limitations. Evaluation using the scale would rely on the print of the scale or light in the screen if used electronically. The best way to measure skin type would be by using a device measuring chromaticity unfortunately such a device was not available in this trial. Ethnicity of the participants are evaluated according to the origin of their parents and this will be used as a proxy of their skin type. The trial is conducted as a single-centre study which leads to both strengths and limitations. A strength is the complete alignment in diagnosing and treatment of the women as they are all followed by the same staff of midwifes and doctors and all give birth at the same labour ward with only a very few exceptions. However, a limitation always related to single-centre studies is the question of whether the study population is representative for a larger population. The trial takes place at a hospital covering both urban and rural areas and people from all socioeconomic groups and represent a broad sample of the entire population in Denmark. Selection bias can of course not be excluded completely. It is possible that people of a lower socioeconomic status with more limited resources might be more prone to decline participation in a clinical trial. On the other hand, it cannot be excluded that the offer of free vitamins will influence the decision towards acceptance of attendance among women with limited economic resources. There is also a risk that we might miss a larger proportion of women with a high interested in health matters e.g. older women who have struggled to become pregnant as these women might wish to remain in full control of their vitamin D intake. However, if the trial shows a beneficial effect of higher doses of vitamin D supplementation, initiated after pregnancy is determined this effect would especially be most beneficial to high risk groups including vulnerable pregnant women with lower socioeconomic status and those who have a higher risk of adverse maternal and neonatal outcomes. As both health care workers and many pregnant women themselves rely on the official recommendations for their health choices in pregnancy, results from this trial can help decision makers to make a new and more accurate recommendation concerning the ideal dose of vitamin D supplementation in pregnancy and in this way reach the majority of the pregnant population.

## Data Availability

The trial is ongoing. We do not have consent from the participants to publish the full dataset, but a de-identified dataset will be available from the corresponding author on reasonable request when the findings from the trial have been published. The results will be presented in international peer reviewed journals and at relevant international conferences.

## Abbreviations

PE: Preeclampsia
GDM: Gestational diabetes mellitus
25(OH)D: 25-Hydroxyvitamin D
SGA: Small for gestational age
FGR: Fetal growth restriction
IUGR: Intrauterine growth restriction
HPLC–MS/MS: High-performance liquid chromatography coupled with tandem mass spectrometry
AGA: Appropriate for gestational age
LGA: Large for gestational age
REDCap: Research Electronic Data Capture
CI: Confidence interval
BMI: Body Mass Index
